# Public Health Benefits from Livestock Rift Valley Fever Control: A Simulation of Two Epidemics in Kenya

**DOI:** 10.1007/s10393-016-1192-y

**Published:** 2016-11-09

**Authors:** Tabitha Kimani, Esther Schelling, Bernard Bett, Margaret Ngigi, Tom Randolph, Samuel Fuhrimann

**Affiliations:** 1 Department of Agricultural Economics & Agribusiness, Egerton University, Njoro, Kenya; 2 International Livestock Research Institute, Nairobi, Kenya; 3 Swiss Tropical and Public Health Institute, Basel, Switzerland; 4 University of Basel, Basel, Switzerland

**Keywords:** public health, benefits, Rift Valley fever, livestock

## Abstract

In controlling Rift Valley fever, public health sector optimises health benefits by considering cost-effective control options. We modelled cost-effectiveness of livestock RVF control from a public health perspective in Kenya. Analysis was limited to pastoral and agro-pastoral system high-risk areas, for a 10-year period incorporating two epidemics: 2006/2007 and a hypothetical one in 2014/2015. Four integrated strategies (baseline and alternatives), combined from three vaccination and two surveillance options, were compared. Baseline strategy included annual vaccination of 1.2–11% animals plus passive surveillance and monitoring of nine sentinel herds. Compared to the baseline, two alternatives assumed improved vaccination coverage. A herd dynamic RVF animal simulation model produced number of animals infected under each strategy. A second mathematical model implemented in R estimated number people who would be infected by the infected animals. The 2006/2007 RVF epidemic resulted in 3974 undiscounted, unweighted disability adjusted life years (DALYs). Improving vaccination coverage to 41–51% (2012) and 27–33% (2014) 3 years before the hypothetical 2014/2015 outbreak can avert close to 1200 DALYs. Improved vaccinations showed cost-effectiveness (CE) values of US$ 43–53 per DALY averted. The baseline practice is not cost-effective to the public health sector.

## Introduction

Rift Valley fever (RVF) is an arthropod-borne viral zoonosis that primarily affects domestic ruminants, humans and some wild animals (OIE 2002, 2007). The RVF virus (RVFV) belongs to the Phlebovirus genus under the Bunyaviridae family. Major RVF epizootics (in livestock) and epidemics (in humans) have occurred in several countries in both Africa and Middle East (Bird et al. 2007). In this paper, the word outbreak is used interchangeably with epidemics or epizootics and it means that reported number of RVF cases in people and livestock is higher than normal. The last two outbreaks in eastern Africa occurred in 1997/1998 and 2006/2007 (Woods et al. 2002; Nguku et al. 2010; Anyangu et al. 2010). The long inter-epidemic/epizootic period (IEP) is attributed to association between the outbreaks and occurrence of El Niño rains. The latter are associated with anomalous warming of sea surface temperatures in the eastern equatorial Pacific and the western equatorial Indian Ocean. The above normal rains that follow the El Nino events cause flooding especially in low-lying areas, favouring the hatching of *Aedes* mosquitoes that transmit RVFV (Linthicum et al. 1991; Diallo et al. 2005).

In livestock, Rift Valley fever outbreaks occur after bites from infected mosquitoes (Linthicum et al. 1985; Davies and Highton 1980). A majority of human infections result from contact with blood or organs of infected animals (WHO 2010; LaBeaud et al. 2008; Sang et al. 2010; Nicholas et al. 2014), while few result from bites by infected mosquitoes. Peaks in human RVF incidences coincide with outbreaks (epizootics) in livestock (Woods et al. 2002; Archer et al. 2013). Impacts of RVF outbreaks go beyond livestock producers to affect public health, other livestock value chain actors and connected sectors of the economy (Swanepoel and Coetzer 2004; ILRI 2008; ROK 2009; Pépin et al. 2010; Rich and Wanyoike 2010).

In managing human RVF, governments seek to optimise health gains by reducing number of human cases, severity or duration of disability and deaths. In the process, budgetary constraints introduce difficult decisions on how to allocate limited resources. Health economists support the decisions by providing data on disease burdens (monetary and non-monetary) as well as cost-effectiveness of control options. Monetary costs include control costs and opportunity costs. Disability adjusted live years (DALYs), a non-monetary measure recommended by World Health Organisation (WHO), reflects premature death and reduced quality of human life (disability) in non-fatal Cases (Murray 1994). One DALY is equal to one lost year of “healthy life”.

Cost-effectiveness analysis helps to prioritise public health sector’s investments allowing decision makers to compare financial costs and gains made or likely to arise from different interventions. Expressed as cost of intervention per DALY averted, WHO sets thresholds based on per capita national incomes (World Health Organisation 2014). An intervention that costs less than three times the national annual per capita GDP is considered cost-effective, whereas one that costs less than once the national annual per capita GDP is considered highly cost-effective. For zoonotic problems such as RVF, gains in human health arise from both animal and public health interventions. Therefore, examining costs and benefits at both levels and in particular benefits to public health sectors arising from animal interventions becomes important. Mostly, zoonotic transmission is animal to human and not the reverse making effective interventions to lie outside public health sector. Assessing costs and benefits of control from a multisectoral perspective facilitates identification of strategies that yield the highest benefits to both sectors. Further, knowledge of distribution of benefits would inform animal control cost sharing between animal and public health sectors.

This cost-effectiveness analysis (CEA) examines impacts of four livestock sector level RVF intervention strategies on public health and identifies those that offer highest benefits to the public health sector.

## Methodology

**Figure 1:**
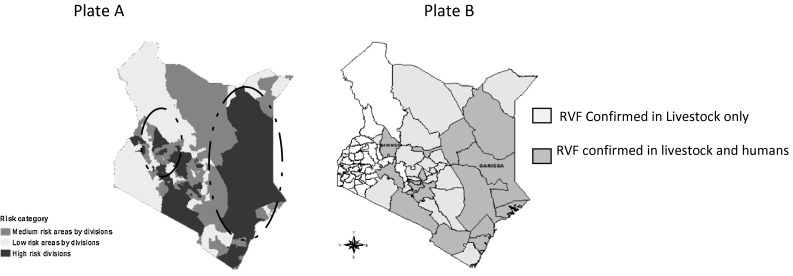
Map of Kenya showing RVF risk status (plate A, source: CDC, Kenya, courtesy of Peninah Munyua) and the 2006/2007 outbreak areas (plate B).

The analysis was limited to RVF high-risk areas in pastoral and agro-pastoral (PAP) livestock systems in Kenya, and for a 10-year period covering two epidemics—the 2006/2007 and a hypothetical one assumed to occur in 2014/2015. Figure 1 plate A shows RVF risk zones in Kenya. The hypothetical 2014/2015 outbreak was assumed to occur in the high-risk areas only. High-risk zones in pastoral and agro-pastoral areas are circled out. Figure 1 plate B shows locations where the 2006/2007 outbreak occurred. The 2006/2007 outbreak represented an actual scenario of without preventive measures: it occurred after a 10-year period during which no measures were applied. The 2014/2015 represented a hypothetical outbreak with control measures. It was assumed to occur after a long inter-epidemic period during which baseline preventive measures were actually applied. The impacts of the baseline measures on the outbreaks were compared to those of alternative measures on the hypothetical 2014/2015 outbreak. In modelling costs and benefits of animal RVF control to public health sector, and for the case of the hypothetical 2014/2015 epidemic, the CEA took a public health partial societal perspective. All significant costs and benefits were considered irrespective of who pays or benefits. The costs of control constituted the numerator in the cost-effectiveness analysis, while outcomes or effectiveness measure was the denominator as cited in Gold et al. (1996).

### Analytical Approach

Seven steps of CEA described in Martins and Rushton (2014) were applied. They are summarised below—though several stages are described together.

#### The Problem, Conceptual Model and Analytical Perspective

As most human RVF cases are transmitted from animals (WHO 2010; LaBeaud et al. 2008; Sang et al. 2010; Nicholas et al. 2014), we assumed that animal RVF control strategies would reduce human epidemics by lowering the number of infected animals and virus amplification cycle in these hosts. Therefore, from a public health perspective, the need for CEA-based prioritisation of animal control measures was considered compelling. Information obtained from the literature and key informants was discussed in two stakeholder workshops. Stakeholders defined four (base strategy and three alternates, Table 1) animal RVF interventions to be subjected to the CEA.

**Table 1:** Description of Four Animal RVF Control Strategies Assessed for Impacts.

Strategy	Inter-epidemic vaccination	Number (millions) of animals that would be 2012–2014 vaccinated			Surveillance option
		Cattle	Sheep and goats	Camels	
Baseline	0^a^	0	2.2	0	0^d^
Alternate 1	1^b^	1.2	4.5	0.6	1^e^
Alternate 2	2^c^	1.7	6.2	0.8	0
Alternate 3	0	0	2.2	0	1

^a^Baseline vaccination of 11% cattle, 1.2% camels and 4.8% sheep and 5.9% goats in 2007; 4.4–8.3% sheep and 6.3–8.3% goats (0% cattle and 0% camels) during the period 2008–2014. The range reflects different proportions in different years, though generated by the model, the rates were informed by the primary data obtained from Ministry in charge of livestock.

^b^Vaccination option 1 comprises baseline vaccination for the period 2007–2011 followed by a shift to annual mass vaccination of 35–43% of all species and ages) in year 2012 and 8–11% of young animals only, in all species in years 2013–2014 The range reflects different proportions in different species and years and were generated by the model.

^c^Vaccination option 2 comprises baseline vaccination for the period 2007–2011 followed by a shift to two annual mass vaccinations of 41–51% and 27–33% (all species, all ages) in years 2012 and 2013, respectively. The range reflects different proportions in different species.

^d^Baseline surveillance option comprises a weak passive surveillance system and 9 sentinel herds monitored three times a year

^e^Enhanced surveillance option, defined as a combination of routine passive enhanced through implementation of a community-based RVF surveillance system and inclusion of vector surveillance activities alongside four times a year wet season sentinel monitoring and epidemiological surveys. Community-based system included (i) a disease community-based control committee with a focal person linked to District Veterinary Office and existing health facility’s public health committees and (ii) a feedback mechanism between field officers and livestock keepers as a key incentive to increase community participation.

The interventions were assumed to be implemented for the period 2007–2014. Both national RVF contingency plan (ROK 2010) and RVF Decision Support (ILRI and FAO 2009) recommend implementation of animal vaccinations and surveillance during the inter-epidemic period in order to minimise impacts of next outbreaks. The rationale lies in the fact that prediction of the 2006/2007 RVF epidemic by NASA was only three months before confirmation of disease in people (Anyamba et al. 2009). A 3-month period is assumed to be insufficient to mount a comprehensive preventive vaccination programme to protect animals. Also, in Kenya, RVF outbreaks occur irregularly: the inter-epidemic period has been 3.6–10 years (Murithi et al. 2011), which complicates timing of measures. The base strategy represented the actual prevailing practice implemented during the period 2007–2011, and was assumed to continue to 2014. Alternates 1 and 2 compared two enhanced vaccination strategies. In Alternate 1, animal vaccination coverage was increased by 460% (four and half times), 67 and 78% in years 2012, 2013 and 2014, respectively, over the base practice (2007–2011). In Alternate 2, it was increased by 512% (five times) and 368% in 2012 and 2013, respectively, over the base strategy. Alternate 3 explored the impacts of enhanced surveillance, assumed to improve early warning and reaction reducing delays in implementing of sanitary bans by 50% (from 4 weeks with baseline surveillance) to 2 weeks. The sanitary bans include bans on movement and marketing of live cattle, sheep, goats and camels and their products. A vaccinated animal was assumed to be protected for life from the disease, reducing the chances of infection and consequent ability to transmit to human being.

#### Modelling

##### Models

First, an individual-based dynamic C++ language with Borland C++ builder 6 model described in detail by Fuhrimann (2011) and highlighted in Zinsstag et al. (2015) was constructed to support simulation of animal outbreaks. The model quantified animal RVF transmission to generate (i) number of cattle, camels, sheep and goats infected during the 2006/2007 and a next hypothetical epidemic in 2014/2015 and (ii) number of animals that died, aborted or well infected and sold or slaughtered. The model represents in a simplified way, livestock dynamics (inflows and outflows disaggregated by species, age and sex categories) during normal and drought periods. The simulation tracked an individual animal over days and years. To observe what happened to the dynamics over the RVF outbreak periods, animals were stratified into susceptible, exposed, infectious and recovered. The impacts of the base and alternate strategies were modelled for the 2014/2015 epidemic period only. To model the impacts of the control strategies on the herd dynamics, assumptions of the biological impacts of the measures on an outbreak were incorporated. For example, vaccinated animals were removed from susceptible populations. The outputs for the hypothetical 2014/2015 outbreak reflected the extent to which the four animal RVF control strategies reduced number of animals infected.

Secondly, a simple compartmental model was developed to simulate human RVFV exposure from infected animals based on the data and parameters outlined in Tables 2 and 3. The model assumed that the human population could be structured into four compartments: Susceptible (S), Exposed (E), Infectious (I) and Recovered/Immune (R).

**Table 2:** Secondary Data on RVFV Infection Levels in Livestock and People, Obtained from Various Publications Documenting RVF Epidemics in Various Countries in Africa.

Variables	Documented RVF epidemic by country and year				
	Kenya 2006/07	Tanzania 2007	Egypt	Mauritania 2003	Mauritania 2010
Total number of livestock in the outbreak sites areas^a^	11,221,797^a^	15,550,052	123,946	7,150,000	775,000
Seroprevalence of RVFV from all the livestock species: cattle, camels, sheep and goats in that order^b^	0.086 0.029 0.138 0.013	0.076 0.049 0.083 –	0.104 0.05 – –	0.16 0.13 0.14 0.33	0.16 0.13 0.14 0.33
Number of livestock infected^c^	1,575,472	1,159.440	8755.604	1,027,180	152,830
Human population in RVF infected areas (number)^d^	1,280,769	10,007,160	655,052	221,301	82,297
Seroprevalence of RVFV in humans (%)	0.13	0.029	0.077	0.03615	0.00039
Infected human population^e^	166,500	291,889	50,439	8000	26,000

^a^In Kenya, the data were derived from the 2009 census, while for the other countries, these data were obtained from FAO (FAOSTAT, 2008).

^b^References used include Nguku et al. (2010), Jost et al. (2010), Munyua et al. (2010) (Kenya), Chengula et al. (2013), Sindato et al. (2011, 2014) (Tanzania), Heinrich et al. (2012), Sumaye et al. (2013), Kamal (2011) (Egypt), Ousmane et al. (2007, 2010) (Mauritania 2003 and 2010 outbreaks) and El Mamy et al. (2011) (Mauritania 2010 outbreak).

^c^These are estimates calculated based on seroprevalence data, except in Kenya, where they were derived from herd dynamics model.

^d^In Kenya, these estimates are based on the 2009 census, while in the other countries, they are derived from United Nations (2015).

^e^The infected human population is derived using the seroprevalence data and human population.

**Table 3:** Description, Values and Sources of the Parameters Used in the Human RVFV Transmission Model.

Symbol	Description	Value	Source
IP	Latent period of RVFV	6	Ikegami and Makino (2011)
RP	Duration of RVF infection	28	Nguku et al. (2010)
mr	Case fatality rate of RVF	0.05	Kahlon et al. (2010) and WHO (2010)
	Duration of the outbreak	90	Jost et al. (2010)

The total human population (N) was represented as follows:

$$ N = S + E + I + R $$

The model was run for a total of 180 days, a duration over which a typical epidemic would take to burn out, with an internal time component of a day. These analyses were carried out using R (version 3.1.1, Dunn 2007) and a system of difference equations used was

1 $$ S_{i + 1} = S_{i} - \left( {S_{i} \times\upbeta \times \frac{{{\text{IL}}_{i} }}{{{\text{TL}}_{i} }}} \right) $$

2 $$ E_{i + 1} = E_{i} + \left( {S_{i} \times\upbeta \times \frac{{{\text{IL}}_{i} }}{{{\text{TL}}_{i} }}} \right) - \left( {E_{i} \times \frac{1}{\text{IP}}} \right) $$

3 $$ I_{i + 1} = I_{i} + \left( {E_{i} \times \frac{1}{\text{IP}}} \right) - \left( {I_{i} \times \frac{1}{\text{RP}}} \right) - \left( {I_{i} \times {\text{mr}}} \right) $$

4 $$ R_{i + 1} = R_{i} + \left( {I_{i} + \frac{1}{\text{RP}}} \right), $$

where β represents the daily transmission rate from livestock to humans, IL represents the infected livestock population (cattle, sheep, goats and camels), TL represents the total livestock population (cattle, sheep, goats and camels), IP represents the latent period of RVFV, RP represents Infectious period of RVFV and mr represents case fatality rate of the disease.

We assumed that all human cases originated from infectious animals during the epidemic and that all animal species were considered to have similar transmission potential of human RVF. The authors appreciated that (i) sheep were infected and had a higher probability of infecting humans and (ii) some infections in humans may result from mosquito bites. However, lack of sufficient data to attribute transmission rates to the different livestock species and mosquitoes leads to a situation where these components of RVFV transmission were ignored. Based on Anyangu et al. (2010), mosquitoes were not significant factors in severe cases of human RVF which carry higher disability weight.

The analyses commenced with the estimation of β for each epidemic presented in Table 2 based on the numbers of human and animal infections, assuming that all the infections in the human populations were acquired from the livestock population over a period of 90 days. This period was fixed based on observations made by Jost et al. (2010). The difference in the cumulative number of infections generated by the model and those observed in the various epidemics (Table 2) was minimised so as to generate epidemic-specific β estimate. This analysis assumed that human exposure occurred when the prevalence of the virus in livestock had achieved an equilibrium level. To simulate a hypothetical RVFV exposure in humans (in 2014), a point β estimate was generated from a uniform distribution, with the outbreak-specific β estimates being used to set the minimum and maximum values of the distribution.

#### Identification and Estimation of Costs

Total monetary public health costs considered included the following:

(i) Ten-year recurrent and fixed expenditures on animal RVF control by public veterinary services and livestock keepers. Since human health benefits from the animal interventions would be produced without separable control costs, we adapted basic elements of joint cost allocation; the “separable cost-remaining benefits” method in multipurpose projects (Gittinger 1982) to allocate the expenditures to both sectors in proportional to benefits gained.
(ii) Household out-of-pocket costs to cater for human cases.
(iii) Direct expenditures by government on diagnosis, treatment and hospitalisation for human inpatients and outpatients.

Costs ii and iii were estimated as a product of number of human cases assumed to be treated and respective unit costs obtained from Schelling and Kimani (2008) and Orinde (2014).

Non-direct recurrent public health sector expenditures and fixed costs for government (salaries, surveillance, deprecation of equipments and transport) could not be estimated due to data and time constraints. The monetary costs and benefits were discounted at 20%.

#### Identification and Determination of Benefit of Control (Outcomes)

Effectiveness of animal interventions from a public health perspective would be measured by the extent to which they reduced both human cases (and therefore DALYs) and case management costs. The DALYs lost during the two human RVF epidemics periods—the 2006/2007 and the hypothetical 2014/2015 (with four strategies)—was estimated. The periods covered November 2006 to June 2007 and November 2014 to June 2015. Animal RVF modelling assumed that there were no animal inter-epidemic transmissions. Hence, inter-epidemic cases were not included. This was informed by lack of sufficient data. Based on Murray (1994), Murray and Lopez (1996) and Narrod et al. (2012), we estimated DALYs as sum of (i) years of healthy life lost (YLL) due to premature death from a standard expected years of life lost (SEYLL) and (ii) for non-lethal cases, years of productive life lived with disease specific disability (YLD). Similar to LaBeaud et al. (2011a), we estimated DALYs for both acute and chronic cases using disability weights of 0.22 and 0.62 and for duration of 0.1 years, respectively, as follows: ${\text{DALYs}} = {\text{YLL}} + \left( {{\text{YLD}}_{\text{acute}} + {\text{YLD}}_{\text{chronic}} } \right)$

$$ {\text{YLL}} = \left( {{\text{Inc}}_{\text{death}} } \right) \times \left( {\text{standard expected years of life lost at median age of death}} \right) $$

$$ {\text{YLD}}_{\text{acute}} = {\text{Inc}}_{\text{acute}} \times {\text{Dw}}_{\text{acute}} \times {\text{Duration}}_{\text{acute}} $$

$$ {\text{YLD}}_{\text{chronic}} = {\text{Inc}}_{\text{chronic}} \times {\text{Dw}}_{\text{chronic}} \times {\text{Duration}}_{\text{chronic}}, $$

where Inc is the Incidence and DW is the disability weight

**Figure 2:**
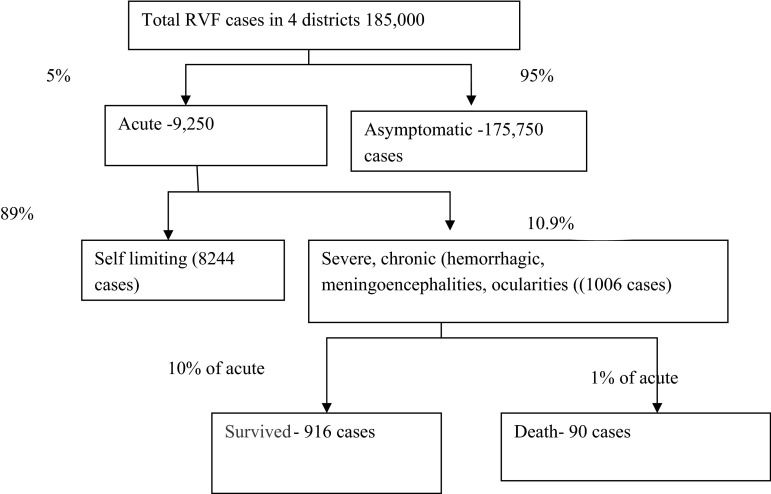
Disaggregated incidence of the RVF cases in RVF hot spots. Total and chronic cases (survived and deaths) were sourced from Nguku et al. (2010); proportions acute, asymptomatic and chronic were informed by Schelling and Kimani (2008), Ikegami and Makino (2011), Nguku et al. (2010), WHO (2010) and Kahlon et al. (2010).

To estimate DALYs lost during the 2006/2007 epidemic, the 13% IgM seroprevalence derived 185,000 human cases reported in Nguku et al. (2010) were assumed to represent all RVF infections in the pastoral and agro-pastoral high-risk areas plus Kilifi district located in mixed farming systems. The latter were excluded from this analysis. Based on the published proportions of underreported, acute, severe and asymptomatic, the cases were disaggregated as shown in Fig. 2. The disaggregation was considered realistic as the 90 deaths documented represent 1% of the estimated acute cases. The range reported in the literature is 0.5–2.0% (LaBeaud et al. 2011a).

Asymptomatic cases were excluded from DALY analysis: they were assumed to result in negligible disability. Demographics' distribution (nine age categories and sex) of confirmed and probable cases reported in Nguku et al. (2010) was extrapolated to all acute and chronic non-fatal cases. Illness during duration of 0.1 years (Orinde 2014) was adopted. Years 2000 (representing 2006) and 2012 (representing 2014) global level highest life expectancy of birth values of 78 years and 82 for men, and 85 and 87 years for women, respectively, were obtained from WHO model life table (WHO 2013). Riou et al. (1989) and LaBeaud et al. (2008) report an upper limit of 4–10% of survivors who develop prolonged ocular and neurological complications of ophthalmitis and meningoencephalitis. Based on this, we assumed the chronic case rate of 10.9% from our data is close to these range.

Similar approach was used to estimate the DALYs associated with the next assumed 2014/2015 RVF outbreak, based on simulated total number of human cases for each control strategy. While discounting of DALYs and age weighting are recommended, this study estimated undiscounted and unweighted values. This is due to the fact that analysis was at a sub-national population, and mainly to rank control strategies. Also, there is increasing critique to discounting and age weighting because the approach values life years lived by people of different ages and generations differently (Anand and Hanason 1997). The benefits were estimated as saved monetary case management costs and DALYs averted.

#### Cost-Effectiveness Analysis and Sensitivity Analysis

Cost-effectiveness of the four animal control strategies was expressed as net present value of public health allocated costs of each control option per DALY averted. Also a benefit cost ratio is computed to compare public health monetary costs and allocated control costs. A discount rate of 20% is used, assuming the base year for evaluating control strategies was 2007. The discount rate was manually varied by 10% for sensitivity analysis.

## Results

### Quantity of Animal Risk Factors for Human RVF Infection During the Two Epidemics

In PAP, animal-related risk factors include drinking raw milk, sheltering livestock, milking animals, disposal of aborted foetuses, assisting animal births, killing or skinning animals, cooking meat and slaughtering animals (Woods et al. 2002; Anyangu et al. 2010; LaBeaud et al. 2008, 2011b); the relative importance of each risk factor behaviour differs. Among the species, sero-positivity association was the greatest with sheep-related activities, followed by goats, cattle and lastly camels. Handling aborted foetuses increased the chance of getting RVF by nearly three times.

The individual livestock dynamic model that derived animal (all species combined) risk load for human transmission is summarised in Table 4. While the numbers of abortions are lower compared to mortality and lactating animals, during the 2006/2007 RVF epidemic, the model estimates close to 1.6 million animals were infected in PAP high-risk areas, while about one thousand (1000) infected animals were sold and slaughtered in slaughter houses located within clean and infected areas and in the process posing a risk to human health. More than 200 infected animals were slaughtered at home.

**Table 4:** Estimated Number of Animal Risk Factors, All Species (Cattle, Sheep, Goats and Camels) Combined, by Epizootic and Control Strategy.

Year of RVF epizootic	Control strategy	Total number of RVF infected animals	Total number of RVF infected animals that or are				
			Aborted	Dead	Sold for commercial slaughter	Slaughtered at home	Lactating
2006/2007	Base	1,575,472	157,866	902,324	1171	231	538,442
Hypothetical 2014/2015	Base	1,744,601	162,302	931,777	8532	1345	814,208
	Alternate 1	1348,598 (23)^a^	129,377 (20)	726,375 (22)	4642 (46)	726 (46)	594,117 (27)
	Alternate 2	1,302,900 (25)	98,579 (40)	698,203 (25)	3893 (54)	678 (50)	586,594 (28)
	Alternate 3	1,743,345 (0)	162,302 (0)	930,535 (0.1)	6509 (24)	1119 (17)	813,273 (0.1)

Source: Computed from the animal RVF transmission model. Livestock start population in 2006/2007 was 11.2 million (combined cattle, sheep, goats, and camels). Start population 2014/2014, was 13.7 million.

^a^Numbers in brackets represent the percentage by which alternate strategies reduce risk load compared to baseline.

For the hypothetical 2014/2015 epidemic, the two alternate strategies with both enhanced vaccination and surveillance (Alternates 1 and 2) reduced the number of infected animals by 23–26% compared to the baseline. Alternate 3 with enhanced surveillance and baseline vaccination reduced the number of infected by less than 1%. Alternates 1 and 2 reduced number of infected animals sold and slaughtered by about a half (46–54%) compared to by less than a quarter (17–24%) in Alternate 3. Overall, Alternates 1 and 2 reduced total risk load by between 27 and 28%, while Alternate 3 reduced total risk load by less than 1%.

### Human Cases During the Hypothetical 2014/2015 RVF epidemic

The daily animal to human transmission coefficients for the different outbreaks was estimated at 0.016% (Mauritania 2003 outbreak), 0.024% (Tanzania), 0.057% (Kenya), 0.068% (Egypt) and 0.1% (Mauritania 2010 outbreak). A random value of 0.069% was obtained by applying a uniform distribution to these values. Estimated human cases transmitted from the infected animals disaggregated by strategy during the hypothetical 2014/2015 epidemic are summarised in Table 5. The base animal control practice resulted in about 158,525 human cases and 78 deaths which are close to total incidence of 2006/2007 in the same area. Alternates 1 and 2 decreased human cases by about a quarter (23% and 25%) compared to baseline.

**Table 5:** Number of Disaggregated Human RVF Cases and Mortality During the Hypothetical 2014/2015 Epidemic Derived from the 0.069% Daily Transmission Rate, Presented by Prevention and Control Options.

Control strategy	Human cases					
	Total	Asymptomatic (95%)	Acute (5%)	Self limiting acute	Chronic	Mortality
Baseline	158,525	150,598	7926	7062	864	78
Alternate 1	122,608	116,478	6130	5462	668	60
Alternate 2	118,461	112,538	5923	5277	646	58
Alternate 3	158,411	150,490	7921	7057	863	78

### DALYs Associated with the RVF Epidemics

Table 6 summarises the undiscounted, unweighted DALY estimates for 2006/2007 RVF epidemic disaggregated by sex and age categories. The total DALY burden for 2006/2007 in PAP high-risk areas was estimated at 3974.05 or 1.50 DALYs per 1000 populations of which mortality contributed to 94.6%. During the hypothetical 2014/2015 epidemic, the baseline animal RVF practice resulted in 4548 DALYs (3.13 per 1000 people) which is 14% higher than DALY burden associated with the 2006/2007. Alternate strategies 1 and 2 showed benefits of averting 1058 and 1187 DALYs, respectively, compared to alternate that averted only 3 DALYs.

**Table 6:** Total DALYs for the 2006/2007 RVF High-risk Areas in PAP.

Sex	Population		DALY per 1000
Sex/age category	Population	DALYs	
Males			
Less than 10 years	542302	3.43	0.01
11–20 years	438,252	579.33	1.32
21–30	164,607	1743.89	10.59
31–40	109,567	325.08	2.97
41–50	80,689	51.98	0.64
51–60	48,455	38.17	0.79
61–70	24,353	79.29	3.26
71–80	8040	7.21	0.90
Over 80	9242	0.86	0.09
Total	1,425,507	2829.25	1.98
Females			
Less than 10 years	485,061	3.43	0.01
11–20 years	320,577	171.01	0.53
21–30	175,934	452.11	2.57
31–40	128,036	401.61	3.14
41–50	64,008	104.65	1.63
51–60	28,028	5.14	0.18
61–70	14,823	4.29	0.29
71–80	6208	0.86	0.14
Over 80	8955	1.71	0.19
Total	1,231,630	1144.80	0.93
Grand total	2,657,137	3974.05	1.50

Source: study computation.

### Public Health Monetary Costs and Benefits Associated with Alternate Animal RVF Control Measures

#### Household Out-of-Pocket and Public Sector Expenditures on Case Management

During the 2006/2007 RVF outbreak, household out-of pocket expenditures on sick in and out patients ranged from US$ 109.6–122.4 (Schelling and Kimani 2008; Orinde 2014), while public hospitals incurred an extra US$ 70.8 per patient on diagnosis, drugs and protective clothing (Schelling and Kimani 2008). We assumed that only severe acute (that progress to chronic) cases (associated with each control strategy) would seek in patient medical treatment. During the hypothetical 2014/2015 epidemic, the number of hospitalised cases would be the highest (864) with base strategy resulting in household out pocket and government direct case management discounted costs of US$ 163,611.6. Households out pocket costs would account for 63.4%.

Compared to the baseline, Alternate strategies 1 and 2 reduced the cost by 30–33%, while Alternate 3 reduced it by 9%. Direct recurrent costs not captured included recruitment of additional staff and staff salary time spent on case management and surveillance. However, during the 2006/2007 epidemic, data obtained from Ministry of Health showed that about US$ 1.3 million from government and unconfirmed amount from Non-Governmental Organisations (NGOs) funded activities. The activities included case management, community education, preventive measures (e.g. vector control and mosquito nets), sampling and transportation of samples, laboratory diagnosis, surveillance and referrals of suspect cases.

#### Animal RVF Control Costs Allocated to Public Health

Table 7 column 2 presents the net present value of 8-year (2008–2015) control costs associated with of the four animal RVF control strategies. The costs were considered as joint costs and allocated to livestock and public heath proportionally to benefit (saved costs). The benefits considered were those related to livestock sector (saved production losses and households (from reduced out-of pocket expense and avoidance of hospitalisation and drugs) and government public heath (saved costs of treatment of sick and hospitalised cases).

**Table 7:** Summary of Net Present Value (NPV) of Costs (US$) and Benefits, and Cost-Effectiveness Analysis (CEA) Parameters of 8-year Animal RVF Control Strategies from a Public Health (PH) Perspective, at Different Discount Rates and for the 2014/2015 Hypothetical Epidemic.

Control strategy	Discount rate 20%								Discount rate 10%
	Animal NPV RVF control costs	Estimated public health NPV monetary costs^a^	NPV Livestock sector (LS) saved costs (avoided production and marketing losses	NPV saved public health monetary costs^b^	DALYs averted	NPV animal prevention costs allocated to PH	US$/DALY averted^c^	Overall benefit (LS and PH)	US$/DALY averted
Baseline	7,122,790	163,713.0							
Alternate 1	10,735,222	114,104	12,699,111	49,609	1,058	50,089	47	12,758,641	77
Alternate 2	10,965,686	109,872	13,771,602	53,841	1,187	51,205	43	13,836,211	62
Alternate 3	8,665,135	148,587	350,637	15,126	3	*		368,789	

* = Alternate 3 is excluded from allocation of costs due to the fact that the strategy had almost similar impacts as the baseline. Year 2007 Exchange rate 66ksh+1 US$.

^a,b^Includes household out-of-pocket and public sector expenditures on case management.

^c^ ***US$*** **=** NPV animal prevention costs allocated to PH. Year for which NPV was estimated, 2007.

Saved public monetary costs (benefits) accounted for 0.5–0.6% (for strategies with improved vaccination, Alternates 1 and 2) and 4.9% (for Alternate 3) of the total public and livestock sectors monetary benefit. Animal health discounted costs allocated to public health were about US$ 50,000–51,000 (Table 7 column 7).

Alternates 2 and 1 returned a control cost per DALY averted of US$62 and US$ 77 and US$43 and US$ 47, respectively, with 10 and 20% discount rate. In 2007 ( assumed decision making year), the per capital GNI was US$ 720 (World Bank 2015). At 20%, the benefit cost ratio (BCR), computed as saved or avoided household out-of-pocket and public sector expenditures on case management, divided by the allocated control costs was about one for both strategies, showing that saved monetary costs are equal to control costs allocated.

## Discussions and Recommendations

This study sought to demonstrate public health sector benefits gained from controlling RVF at animal level. Due to unavailability of animal–human RVF transmission model at the time, we applied two separate models linked through data. The results showed significant public health sector monetary and non-monetary burden (DALYs) associated with the 2006/2007 RVF epidemic in high-risk areas in PAP systems. Considering that the systems carry 53, 66, 73 and 99.7% of the cattle, sheep, goat and camels found in high-risk areas, and that human transmission is mostly through animal contact, the DALYs estimated could constitute a large proportion of the national burden.

For the same epidemic, our estimates of 3974.05 DALYs are lower than higher estimates of 4035.6 reported in Orinde (2014). The difference lies in the data used. Orinde used a disability weight of 0.652 for all cases, and only considered line listed cases and human population in only three Counties. Nguku et al. (2010) report that not all line listed cases were due to RVF. Our study considered human population in all RVF high-risk areas in PAP system, and used prevalence-derived incidence to accommodate for under reporting. Both studies imply that the national burden of RVF associated with 2006/2007 RVF outbreak might be higher than the estimates. The animal–human RVF transmission modelling showed that under the animal control base strategy, the magnitude of a next hypothetical epidemic would be nearly similar.

Total DALYs associated with the 2006/2007 and the hypothetical 2014/2015 (under base strategy) translate to 852 annual unweighted, undiscounted DALYs, that represent 7% of the upper limit and more than twice the lower limit of the global RVF burden reported in LaBeaud et al. (2011a). Inherent in DALY estimation process, it is the YLL from RVF mortality that accounts for the largest proportion of DALYs estimated.

Based on WHO thresholds for cost-effectiveness (WHO 2013) and compared to baseline improved vaccination coverage in camels and cattle from 0% to between 7 and 51% (depending on species, age targeted and strategy), and sheep and goats (1–2 fold) 2 years before an RVF epidemic can be considered to be highly cost-effective from a public health perspective: in terms of reduction in DALYs and direct treatment costs for human cases. Under base practice, only 4–9% small ruminants were annually vaccinated for 7 years before the hypothetical 2014/2015 outbreak. World Health Organisation’s Choosing Interventions that are Cost-Effective (WHO-CHOICE) project indicates that an intervention with a cost per DALY averted that is less than three times the national annual GDP per capita is considered cost-effective, whereas one that costs less than once the national annual GDP per capita is considered highly cost-effective. The strategies can significantly (23–26%) reduce DALYs. The base practice is not cost-effective for the public health sector. Also, the benefit cost ratio that compared allocated costs to saved monetary costs shows that compared to baseline, the two alternate strategies with enhanced vaccination had a BCR of about one, while Alternate 3 had less than 1. Higher or equal monetary benefits over allocated costs reflect additional benefits to the DALYs averted.

Based on our models, enhancing surveillance while keeping vaccination level at base would yield only small benefits to the public health sector. However, effective animal surveillance systems would allow public health sectors to implement early public health communication to minimise contact with infected animals. Such benefits were not captured in our modelling. Enhanced animal surveillance also supports earlier implementation of livestock sanitary bans; and therefore, potential contacts through slaughter and marketing activities.

However, the results must be interpreted from a perspective of that in modelling, animal–human transmission, this study faced challenges as no animal–human transmission model had been developed to support multisectoral analysis. At the same time, few datasets on joint animal and public health outbreak investigations existed. This study, therefore, relied on few data points from five epidemics in four countries. Similar difficulties were reported by other similar studies (LaBeaud et al. 2011a; Randi 2011), to an extent that the latter assumed that, in case of an incursion, human RVF outbreak in Southeast Texas, USA, would acquire spread and infection rates similar to West Nile Virus. On the other hand, LaBeaud et al. (2011a) presented annual global burden of RVF as a range of 353–11,958. In addition, while animal transmission model estimated the number of animals that would abort, be lactating or infected and slaughtered during the outbreak in Kenya, lack of similar data for the other four outbreaks in other countries denied the authors an opportunity to modifying the transmission based on relative risk. Further, due to the same data and modelling challenges, we combined all animal species data which make it hard to tease apart the relative contribution from different risk factors such as drinking un-boiled milk as fewer people would drink raw milk from sheep, goats and cattle compared to camels.

To overcome these challenges, and particularly to strengthen One Health economic analysis of zoonotic diseases, there is a need for future joint epidemiological investigations to generate data to support animal–human RVF epidemiological modelling. Also, there is need for public health studies that estimate (i) the relative contribution of different public health measures such as surveillance and communication to the outcome of the epidemic and (ii) animal–human contact rates and transmission probabilities. Generating longitudinal data on human and livestock cases during both the epidemics and inter-epidemic and scale of measures applied would support modelling of livestock-human transmission as was the case of brucellosis modelling in Mongolia in Roth et al. (2003).

Further, in modelling the magnitude of the hypothetical 2014/2015 RVF epidemic, we assumed that changes in human behaviour prior to and during epidemics would not change and therefore, the same force of infection is maintained. This was influenced by observations that the PAP areas are under developed, and receive relatively lower quantity and quality of health services including community-based communication for behaviour change. Consequently, therefore, incidences of zoonosis are higher than in other farming systems as shown in the case of brucellosis (Regassa et al. 2009; Racloz et al. 2013) and anthrax (Nkedianye and Herrero 2007). Some risk factors for human brucellosis and anthrax such as living close proximity to livestock, handling livestock and consumption of raw products are similar to those of RVF. Owange et al. (2014) highlight pastoralist’ perception where mosquito bites are perceived as the key risk factors compared to contact with infected livestock and livestock products which are contrary to other studies (Woods et al. 2002; Anyangu et al. 2010). Finally, we note that errors could have resulted from the modelling process where two models are used to arrive at this cost-effectiveness analysis. The errors could have made the model less sensitive to changes in some of the key processes being studied.

While the results show that increasing peace time animal vaccination coverage reduces the magnitude of human outbreaks, the baseline practice shows that national governments seem to find it difficult to achieve good coverage levels as the risk is perceived to be low. While new vaccines are being developed including multivalent ones that might be possible to be applied more frequently alongside those of other diseases, vaccination coverage for all diseases is considered to be lower than expected. A better strategy—e.g. routine vaccination at a given coverage and a reactive vaccination when a risk warning is given to shore-up the desired levels of coverage—is required. Despite that enhanced surveillance is expected to reduce potential number of human cases following early implementation of sanitary bans, the modelling failed to capture this dynamics. Therefore, the real value of animal surveillance in terms of reducing human could not be explicitly explained. To better respond to future outbreaks, contingency plans and decision support tools are suggesting more pragmatic efforts of implementing surveillance.

## References

[CR2] Anand S, Hanson K (1997). Disability-adjusted life years: a critical review. Journal of Health Economics.

[CR56] Anyamba A, Chretien J-P, Small J, Tucker CJ, Formenty PB, Richardson JH, Britch SC, Schnabel DC, Erickson RL, Linthicum KJ (2009). Prediction of a Rift Valley fever outbreak. Proceedings of the National Academy of Sciences of the United States of America.

[CR1] Anyangu AS, Gould LH, Shahnaaz K, Sharif SK, Nguku PM, Omolo JO, Mutonga D, Rao CY, Ledeman ER, Schnabel D, Paweka JT, Katz M, Hightower AM, KaruikiNjenga MK, Daniel R, Feikin DR, Breiman RF (2010). Risk factors for severe Rift Valley fever infection in Kenya. American Journal of Tropical Medicine and Hygiene.

[CR3] Archer BN, Thomas J, Weyer J, Cengimbo A, Landoh DE, Jacobs C, Ntuli S, Modise M, Mathonsi M, Mashishi MS, Leman PA, Roux C, Vuren PJ, Kemp A, Paweska J, Blumberg L (2013) Epidemiologic investigations into outbreaks of Rift Valley fever in humans, South Africa, 2008–2011. *Emerging Infectious Diseases* 19(12):1918–1925. www.cdc.gov/eid 10.3201/eid1912.121527 PMC3840856 29360021

[CR5] Bird BH, Khristova ML, Rollin PE, Ksiazek TG, Nichol ST (2007). Complete genome analysis of 33 ecologically and biologically diverse Rift Valley fever virus strains reveals widespread virus movement and low genetic diversity. Journal of Virology.

[CR57] Chengula AA, Mdegela RH, Kasanga CJ (2013) Socio-economic impact of Rift Valley fever to pastoralists and agro pastoralists in Arusha, Manyara and Morogoro regions in Tanzania. SpringerPlus 2:549. doi:10.1186/2193-1801-2-549 10.1186/2193-1801-2-549 PMC3825084 24255846

[CR6] Davies FG, Highton RB (1980). Possible vectors for Rift Valley fever in Kenya. Transactions of the Royal Society of Tropical Medicine and Hygiene.

[CR7] Diallo M, Nabeth P, Ba K, Sall AA, Ba Y, Mondo M (2005). Mosquito vectors of the 1998–1999 outbreak of Rift Valley Fever and other arboviruses (Bagaza, Sanar, Wesselsbron And West Nile) In Mauritania and Senegal. Medical and Veterinary Entomology.

[CR8] Dunn K (2007) Tweedie exponential family models. R. 2007

[CR9] Food and Agriculture Organization of the United Nations (2008) FAOSTAT statistics database. Rome

[CR10] Fuhrimann S (2011) Rift Valley fever in Kenyan pastoral livestock. Master Thesis submitted to the University of Basel

[CR12] Gittinger JP (1982). Economic Analysis of Agricultural Projects.

[CR13] Gold MR, Siegel JE, Russell LB, Weinstein MC (eds) (1996) *Cost-Effectiveness in health and medicine*. New York: Oxford University Press

[CR14] Heinrich N, Saathoff E, Weller N, Clowes P, Kroidl I, Ntinginya E, Machibya H, Maboko L, Loscher T, Dobler G, Hoelscher M (2012). High seroprevalence of Rift Valley fever and evidence for endemic circulation in Mbeya region, Tanzania, in a cross-sectional study. PLoS Neglected Tropical Diseases.

[CR16] Ikegami T, Makino S (2011) The pathogenesis of Rift Valley fever. *Viruses* 3(5):493–519. doi:10.3390/v3050493 10.3390/v3050493 PMC3111045 21666766

[CR15] ILRI (2008) Learning the lessons of Rift Valley fever: improved detection and mitigation of outbreaks—participatory assessment of Rift Valley fever surveillance and rapid response activities. ILRI (unpublished report)

[CR700] ILRI, FAO (2009) Decision-support tool for prevention and control of Rift Valley. Fever epizootics in the Greater Horn of Africa. Version I. *ILRI Manuals and Guides*. no. 7. 28 p. Nairobi (Kenya): ILRI 10.4269/ajtmh.2010.83s2a03 PMC2913494 20682910

[CR58] Jost CC, Nzietchueng S, Kihu S, Bett B, Njogu G, Swai ES, Mariner JC (2010). Epidemiological assessment of the Rift Valley fever outbreak in Kenya and Tanzania in 2006 and 2007. American Journal of Tropical Medicine and Hygiene.

[CR18] Kamal SA (2011) Observations on rift valley fever virus and vaccines in Egypt. *Virology Journal* 8:532. http://www.virologyj.com/content/8/1/532. Accessed 3 July 2014 10.1186/1743-422X-8-532 PMC3264540 22152149

[CR17] Kahlon SS, Peters CJ, LeDuc J, Muchiri EM, Muiruri S, Kariuki Njenga M, Breiman RF, White AC, King CH (2010). Severe Rift Valley fever May present with a characteristic clinical syndrome. The American journal of tropical medicine and hygiene.

[CR21] LaBeaud AD, Muchiri EM, Ndzovu M, Mwanje MT, Muiruri S, Peters JC, King CH (2008). Interepidemic Rift Valley fever virus seropositivity, North-eastern Kenya. Emerging Infectious Diseases.

[CR19] LaBeaud AD, Bashir F, King CH (2011a) Measuring the burden of arboviral diseases: the spectrum of morbidity and mortality from four prevalent infections. *Population Health Metrics* 9(1):2014. doi:10.1186/1478-7954-9-1 10.1186/1478-7954-9-1 PMC3024945 21219615

[CR20] LaBeaud AD, Muiruri S, Sutherland LJ, Dahir S, Gildengorin G, Morrill J, Muchiri EM, Peters CJ, King CH (2011b) Postepidemic analysis of Rift Valley fever virus transmission in North-eastern Kenya: a village cohort study. *PLoS Neglected Tropical Diseases* 5:e1265. Accessed 4 July 2014 10.1371/journal.pntd.0001265 PMC3156691 21858236

[CR22] Linthicum KJ, Bailey CL, Tucker CJ, Angleberger DR, Cannon T, Logan TM, Gibbs PH, Nickeson J (1991). Towards real-time prediction of Rift Valley fever epidemics in Africa. Preventive Veterinary Medicine.

[CR23] Linthicum KJ, Davies FG, Kairo A, Bailey CL (1985). Rift Valley fever virus (family Bunyaviridae, genus *Phlebovirus*): isolations from *Diptera* collected during an interepizootic period in Kenya. Journal of Hygiene.

[CR24] Mamy ABO, Mohamed OB, Yahya B, Katia I, Mamadou LD, Ba H, Mamadou YD, Mohamed OBK, Diop M, Modou M, Yaya T, Bengoumi M, Puech L, Ludovic P, Filip C, Rocque S, Doumbia B (2011) Unexpected Rift Valley fever outbreak, northern Mauritania. *Emerging Infectious Diseases* 17(10):1894–1896. http://wwwnc.cdc.gov/eid/article/17/10/pdfs/11-0397.pdf. Accessed 20 May 2014 10.3201/eid1710.110397 PMC3310676 22000364

[CR25] Martins SB, Rushton J (2014) Cost-effectiveness analysis: adding value to assessment of animal health, welfare and production. *Revue scientifique et technique (International Office of Epizootics)* 33(3):681–689 10.20506/rst.33.3.2312 25812198

[CR59] Munyua P, Murithi RM, Wainwright S, Githinji J, Hightower A, Mutonga D, Macharia J, Ithondeka PM, Musaa J, Breiman RF, Bloland P, Njenga MK (2010). Rift Valley fever outbreak in livestock in Kenya, 2006–2007. The American Journal of Tropical Medicine and Hygiene.

[CR60] Murithi RM, Munyua P, Ithondeka PM, Macharia JM, Hightower A, Luman ET, Breiman RF, Njenga MK (2011). Rift Valley fever in Kenya: history of epizootics and identification of vulnerable districts. Epidemiology and Infection.

[CR26] Murray CJ (1994). Quantifying the burden of disease: the technical basis for disability-adjusted life years. Bulletin of the World Health Organization.

[CR27] Murray CJ, Lopez AD (1996) *The Global Burden of Disease: A Comprehensive Assessment of Mortality and Disability from Diseases, Injuries, and Risk Factors in 1990 and Projected to 2020*. Harvard University Press

[CR28] Narrod C, Zinsstag J, Tiongco M (2012) A One Health framework for estimating the economic costs of zoonotic diseases on society. *EcoHealth* 9:150–162. doi:10.1007/s10393-012-0747-9 10.1007/s10393-012-0747-9 PMC3415616 22395956

[CR30] Nguku PM, Sharif SK, Mutonga D, Amwayi S, Omolo J, Mohammed O, Farnon EC, Gould LH, Lederman E, Rao C, Sang R, Schnabel D, Feikin DR, Hightower A, Njenga MK, Breiman RF (2010). An investigation of a major outbreak of Rift Valley fever in Kenya: 2006–2007. American Journal of Tropical Medicine and Hygiene.

[CR31] Nicholas DE, Jacobsen KH, Waters NM (2014). Risk factors associated with human Rift Valley fever infection: systematic review and meta-analysis. Tropical Medicine & International Health.

[CR4] Nkedianye BC, Herrero MD (2007). Maasai perception of the impact and incidence of malignant catarrhal fever MCF in southern Kenya. Preventive Veterinary Medicine.

[CR33] OIE (2002) Rift Valley fever. Retrieved June 2010, from World Organisation for Animal Health: http://www.oie.int/eng/maladies/fiches/a_A080.htm. Accessed Feb 2012

[CR32] OIE (2007) The OIE rallies countries for combating Rift Valley fever in Africa and the middle East—press release. http://www.oie.int/eng/press/en_070619.htm. Accessed 21 Apr 2009

[CR34] Orinde AB (2014) *Quantifying the Burden of Rift Valley Fever in Humans Using Disability Adjusted Life Years, Kenya*. Master’s Thesis submitted to Jomo Kenyatta University

[CR36] Ousmane F, Mawlouth D, Djibril D, Bezeid O, Hampathé B, Mbayame N, Ibrahima D, Sid AOM, Kader N, Diawo D, Peinda OL, Boubacar D, Pierre N, François S, Baïdy L, Ousmane MD (2007) Rift Valley fever outbreak with East-Central African Virus Lineage in Mauritania, 2003. *Emerging Infectious Diseases* 13(7):1016–1023. www.cdc.gov/eid 10.3201/eid1307.061487 PMC2878230 18214173

[CR35] Ousmane F, Hampathé B, Yamar B, Caio CMF, Oumar F, Oumar N, Isselmou OE, Paolo MAZ, Mawlouth D, Amadou AS (2010) Re-emergence of Rift Valley Fever, Mauritania. *Emerging Infectious Diseases* 20(2). doi:10.3201/eid2002.130996. (www.cdc.gov/eid. Accessed Feb 2014

[CR29] Owange NO, Ogara WO, Kasiiti J, Gathura PB, Okuthe S, Sang R, Affognon H, Onyango-Ouma W, Landmann TT, Mbabu M (2014) Perceived risk factors and risk pathways of Rift Valley fever in cattle in Ijara district, Kenya. *The Onderstepoort Journal of Veterinary Research* 81(1). doi:10.4102/ojvr.v81i1.780. Accessed 20 Nov 2014 10.4102/ojvr.v81i1.780 25686079

[CR37] Pépin M, Pléé L, Lancelot R (2010). Rift Valley fever—a threat for Europe?. Euro surveillance.

[CR38] Racloz V, Schelling E, Chitnis N, Roth F, Zinsstag J (2013). Persistence of brucellosis in pastoral systems. Revue scientifique et technique (International Office of Epizootics).

[CR39] Randi CH (2011) Assessment of U.S. agriCulture Sector and Human Vulnerability to a Rift Valley Fever Outbreak. Msc Thesis Submitted to the Office of Graduate Studies of Texas A&M University

[CR40] Regassa G, Mekonnen D, Yamuah L, Tilahun H, Guta T, Gebreyohannes A, Aseffa A, TAbdoel TH, Smits HL (2009) Human brucellosis in traditional pastoral communities in Ethiopia. *International Journal of Tropical Medicine* 4(2):59–64. http://medwelljournals.com/abstract/?doi=ijtmed.2009.59.64. Accessed 4 Aug 2014

[CR41] Rich KM, Wanyoike F (2010). An assessment of the regional and national socio-economic impacts of the 2007 Rift Valley fever outbreak in Kenya. American Journal of Tropical Medicine and Hygiene.

[CR42] Riou O, Philippe B, Jouan A, Coulibaly I, Mondo M, Digoutte JP (1989). Neurologic and neurosensory forms of Rift Valley fever in Mauritania. Bulletin de la Societe de pathologie exotique et de ses filiales.

[CR43] ROK (2009) *Socio-Economic Assessment of the 2006/2007 Rift Valley Fever*. Unpublished report of the Ministry of Livestock Development, Department of Veterinary Services

[CR620] ROK (2010) Contingency Plan for Rift Valley Fever. *A Final draft document of the Ministry of Livestock Development*, Department of Veterinary Services

[CR44] Roth F, Zinsstag J, Orkhon D, Chimed-Ochir G, Hutton G, Cosivi O, Carrin G, Otte J (2003). Human health benefits from livestock vaccination for brucellosis: case study. Bulletin of the World Health Organization.

[CR45] Sang R, Kioko E, Lutomiah J, Warigia M, Ochieng C, O’Guinn M, Lee S, Koka H, Godsey M, Hoel D, Hanafi H, Miller B, Schnabel D, Breiman R, Richardson J (2010). Rift Valley fever virus epidemic in Kenya, 2006/2007: the entomologic investigations. The American journal of tropical medicine and hygiene.

[CR46] Schelling E, Kimani T (2008) Human and animal health response capacity and costs: a rapid appraisal of the 2007 Rift Valley fever outbreak in Kenya. An ILRI unpublished report

[CR61] Sindato C, Karimuribo E, Mboera LEG (2011) The epidemiology and socio-economic impact of rift valley fever epidemics in Tanzania: a review. *Tanzania Journal of Health Research* 13(Suppl 1):305–318. doi:10.4314/thrb.v13i5.1 10.4314/thrb.v13i5.1 26591986

[CR47] Sindato C, Karimuribo ED, Pfeiffer DU, Mboera LEG, Kivaria F, Dautu G, Bett B, Paweska JT (2014). Spatial and temporal pattern of Rift Valley Fever outbreaks in Tanzania; 1930 to 2007. PLoS One.

[CR48] Sumaye RD, Geubbels E, Mbeyela E, Berkvens D (2013). Inter-epidemic transmission of Rift Valley fever in livestock in the Kilombero River valley, Tanzania: a cross-sectional survey. PLoS Neglected Tropical Diseases.

[CR49] Swanepoel R, Coetzer JAW (2004) Rift Valley fever. In: JAW Coetzer, RC Tustin (eds) *Infectious Diseases of Livestock with Special Reference to Southern Africa.* Cape Town: Oxford University Press, pp 1037–1070, Epizooties, 24(3), 847–856. Epizooties, Oxford University Press

[CR50] United Nations (2015) *World Population Prospects, the 2015 Revisions*. United Nations, Department of Economic and Social Affairs, Population Divisions

[CR53] WHO (2010) *Rift Valley Fever.* Fact sheet N°207 Revised May 2010. http://www.who.int/mediacentre/factsheets/fs207/en/. Accessed 1 Sept 2012

[CR52] WHO (2013) *Model Life Table.* http://apps.who.int/gho/data/node.main.688. Accessed 9 May 2014

[CR54] Woods CW, Karpati AM, Grein T, McCarthy N, Gaturuku P, Muchiri E, Dunster L, Henderson A, Khan AS, Swanepoel R, Bonmarin I, Martin L, Mann P, Smoak BL, Ryan M, Ksiazek TG, Arthur RR, Ndikuyeze A, Agata NN, Peters CJ, The World Health Organization Hemorrhagic Fever Task Force (2002) An outbreak of Rift Valley fever in North-eastern Kenya. *Emerging Infectious Disease* 8(2):138–144 10.3201/eid0802.010023 PMC2732454 11897064

[CR55] World Bank (2015) http://data.worldbank.org/indicator. Accessed April 2015

[CR51] World Health Organization (2014) *Choosing Interventions that are Cost-Effective [Internet]*. Geneva: World Health Organization. http://www.who.int/choice/en/. Accessed 3 May 2015

[CR11] Zinsstag J, Fuhrimann S, Hattendorf J, Chitnis N (2015) Animal-human transmission models. In: *One Health. Theory to practice. Integrated Health Approaches*. London: CAB International, pp 122–133

